# Human host determinants influencing the outcome of *Trypanosoma brucei gambiense* infections

**DOI:** 10.1111/j.1365-3024.2011.01287.x

**Published:** 2011-08

**Authors:** B Bucheton, A MacLeod, V Jamonneau

**Affiliations:** 1 Institut de Recherche pour le Développement (IRD), Unité Mixte de Recherche IRD-CIRAD 177, Campus International de BaillarguetMontpellier, France; 2 Centre International de Recherche-Développement sur l’Elevage en zones Subhumides (CIRDES), Unité de recherches sur les bases biologiques de la lutte intégréeBobo-Dioulasso, Burkina Faso; 3 Wellcome Trust Centre for Molecular Parasitology, College of Medical, Veterinary and Life Sciences, Sir Henry Wellcome Building for Comparative Medical SciencesGlasgow, UK

**Keywords:** asymptomatic carriers, epidemiology, genetic factors, Human African trypanosomiasis, *Trypanosoma brucei gambiense*, trypanotolerance

## Abstract

Since first identified, human African trypanosomiasis (HAT) or sleeping sickness has been described as invariably fatal. Increasing data however argue that infection by *Trypanosoma brucei gambiense*, the causative agent of HAT, results in a wide range of outcomes in its human host and importantly that a number of subjects in endemic areas are apparently able to control infection to low levels, undetectable by the classical parasitological tests used in the field. Thus, trypanotolerance seems to occur in humans as has already been described in cattle or in the rodent experimental models of infection. This review focuses on the description of the diversity of outcomes resulting from *T. b. gambiense* in humans and on the host factors involved. The consequences/impacts on HAT epidemiology resulting from this diversity are also discussed with regard to implementing sustainable HAT control strategies.

## HAT: The Epidemiology of Disease is Still Unclear

### Lessons from a century of history

Human African trypanosomiasis (HAT) or sleeping sickness is a disease of sub-Saharan Africa caused by two sub-species of trypanosome transmitted by tsetse flies, *Trypanosoma brucei gambiense* (in West and Central Africa) and *Trypanosoma brucei rhodesiense* (in East and South Africa), with *T. b. gambiense* causing >90% of all cases (1). Importantly, HAT has emerged/re-emerged in the last century throughout most of sub-Saharan Africa. Whereas hundreds of thousands of cases occurred in sub-Saharan Africa in the early part of the 20th century as the result of exploiting tsetse-infested areas by the colonial administrations, systematic screening and treatment of millions of individuals led to transmission coming near to a halt by the 1960s. However, the disease progressively flared up since the 1970s and returned to alarming levels with an estimation in the range of 300 000–500 000 infected people at the end of the last century (2,3). Faced with this situation, an intensification of control efforts was conducted by National Control Programmes (NCP) with substantial financial and technical support from WHO, bilateral cooperation and NGOs. Current results are promising, and WHO reported a significant decline in the number of new cases. Out of the 36 endemic countries, 20 are close to achieving the target of reporting no new cases and eight reported <100 new cases per year (1). Thus, elimination has become again a feasible objective in many endemic countries (4). This is particularly true in West Africa where the disease has progressively disappeared from the savannah areas following active surveillance and treatment that considerably reduced the human reservoir of the parasite from the 1950s to the 1960s (5). The halt of *T. b. gambiense* transmission in these areas is presumably also attributable to a concomitant decrease in host–vector contacts that are related to several factors such as climatic changes, population growth leading to the development of hydrological structures reducing contacts occurring during watering activities and man-made environmental degradation of *Glossina palpalis gambiensis* (the main vector of *T. b. gambiense* in West Africa) biotopes along river banks (6–8). The decrease in tsetse–human contacts is also probably in part responsible for the fact that HAT has not re-emerged, particularly in the historical foci of South-west Burkina Faso. This region was faced with a massive return of repatriates coming mostly from active HAT foci in Côte d’Ivoire during the political crisis that occurred in this country. Despite this apparently favourable context, medical surveys performed in 2006–2007 failed to detect any return of the disease in these areas (9).

Since the 1980s, control of *T. b. gambiense* HAT is highly dependent on mass screening of the population by the card agglutination test for trypanosomiasis [CATT, (10)], which identifies individuals with antibodies to *T. b. gambiense*. The blood, lymph and cerebrospinal fluid (CSF) of individuals positive for this serological test are then examined by microscopy (11). All individuals positive by both CATT and microscopy are treated to clear the disease and to reduce the parasite reservoir and thus lower or disrupt transmission (12,13). This strategy resulted in a 69% reduction in the number of new cases reported during the period 1997–2006 in *T. b. gambiense* endemic areas (1). However, field observations in current HAT active foci where environmental conditions are still favourable for transmission, for example the Guinean mangrove foci (14) and in the mesophilic forests foci of Côte d’Ivoire (15), have shown that while this strategy appeared to be efficient in lowering disease prevalence, the disease remains present in many areas despite repeated active surveillance and treatment. In the context of disease elimination, it is therefore crucial to obtain a better understanding of the reasons underlying disease re-emergence or maintenance to insure the sustainable control or elimination of HAT. Whereas political instability, population movements combined with decreased awareness and shortage of funds are often put forward and indubitably play an important role (5), other biological factors such as genetic variation in host and parasite, to which little attention is paid, may also be involved to account for the re-emergence/maintenance of HAT in historical foci.

### Uncertainties remain in the transmission of *Trypanosoma brucei gambiense*

The observations mentioned above concerning the maintenance and/or the re-emergence of HAT in a given area suggest that a hitherto unknown reservoir of *T. b. gambiense* parasites exists and takes part in transmission in addition to patients with HAT towards which control programmes are targeted. This assumption has recently been put forward by a parasite population genetic study carried out in the HAT foci of Guinea and Côte d’Ivoire (16). Results of this study indicated that the estimated parasite effective clonal population size appeared to be higher than the corresponding observed local prevalence of HAT determined by mass screening of the population. Thus, at least part of the parasite population seems to be unaccounted for. For *T. b. gambiense*, the transmission cycle is mostly thought to be human to human; however, several studies have shown that wild and domestic animals (17–22) could be infected by *T. b. gambiense*, which could act as animal reservoirs of disease. However, the role played by these animal reservoirs in parasite transmission to humans is yet unclear (i.e. whether they increase the transmission to human or on the contrary act as bait thus providing some degree of protection). This needs to be further investigated (23). Another possibility would be that human asymptomatic carriers exist but that their parasitaemia is so low that the parasites are undetectable by classical parasitological methods used in the field. The existence of such individuals has been shown in many infectious diseases for example by HIV controllers (24) or in *Leishmania infantum* (another trypanosomatid) where asymptomatic carriers have been identified by parasite-specific antibodies or DNA found in an important fraction of healthy blood donors in the Mediterranean area (25,26). On the contrary, infection by *T. b. gambiense* is still largely considered as being 100% fatal if untreated. However, both old field observations (27) and more recent epidemiological studies (summarized below) now provide a bulk of evidence showing this is not the case. Human infections by *T. b. gambiense* appear to be complex, governed by the interaction of environmental, parasite and host factors leading to a great diversity of infection outcomes (28).

## Variation in *Trypanosoma brucei gambiense* Infection Outcomes

### Variation in disease severity

**Figure 1:**
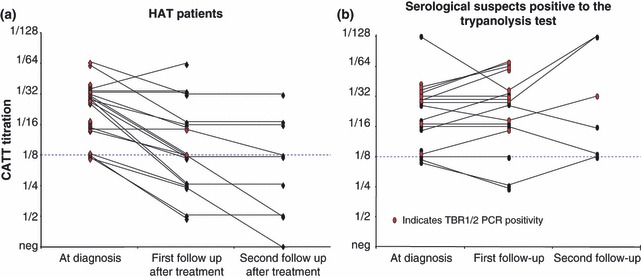
Follow-up of patients and serological suspects in Guinea. Trypanolysis positive serological suspects (b) remained with high CATT titers during their follow-up and parasite DNA was detected by PCR in half of them. All together, the presence of parasite DNA and the high and long lasting CATT reactivity observed in these subjects, in contrast to what is observed in treated patients (a), strongly suggests that these individuals are asymptomatic carriers of *T. b. gambiense* with low blood parasitemia. Follow-up visits were made at six month intervals.

**Figure 2:**
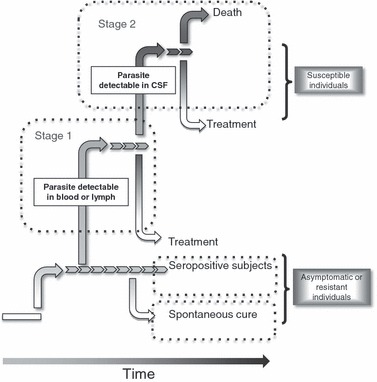
Diversity of *Trypanosoma brucei gambiense* infection outcomes in HAT endemic areas. The accepted view of trypanosome infections is that the disease progresses from stage 1 to stage 2 disease over time. However long-term seropositive have been identified and recently we have identified that some of these seropositive individuals have self-cured. Seropositive individuals are asymptomatic and can be considered tolerant whereas those that self-cured may be considered resistant.

HAT caused by *T. b. gambiense* is classically described as a chronic disease characterized by an early haemolymphatic phase (first stage) associated with nonspecific symptoms such as intermittent fevers and headaches, followed by a meningoencephalitic phase (second stage) where the parasite invades the central nervous system leading to neurological disorders and death if left untreated. Because they constitute a source of parasite for tsetse transmission and because of the toxicity of the second-stage-specific treatment (29), patients with HAT are treated as soon as possible after diagnosis. In *T. b. rhodesiense* infections, an inoculation ‘chancre’ is often observed at the site of the bite by the infected tsetse, which can be used to estimate disease duration (30,31). However, the inoculation chancre is rare in *T. b. gambiense* HAT, and combined with the fact that first-stage symptoms are often mild and not specific, it is almost impossible to have access to reliable data on disease duration and so the natural progression of *T. b. gambiense* infection has not yet been fully characterized in humans (27). However in *T. b. gambiense*, first-stage disease seems to be highly variable between individuals, and several studies have reported acute forms, progressing rapidly to the second stage (32,33) while more often the first stage can last from several months to years (34). Importantly, a recent survey based on the long-term follow-up of HAT patient refusing treatment in Côte d’Ivoire enabled the identification of subjects that were initially diagnosed in first stage by microscopy, yet on follow-up examination had no detectable clinical symptoms and no detectable parasitaemia by microscopy. After long-term follow-up, a drop in antibody titres to seronegative levels was detected in some of these subjects, indicating that they have self-cured. On the contrary, others maintained a long-lasting serological response, suggesting that these individuals were able to control blood parasitaemia to levels by undetectable by microscopy and may thus be considered as asymptomatic carriers of parasites (34–36). Whereas self-cure may be regarded as a rare phenomenon, individuals displaying high serological responses but negative by microscopy are frequently encountered in the field during medical surveys. These individuals have been termed seropositives. Management of these individuals is still a controversial issue. Whereas it has been recommended to treat seropositive subjects with CATT end titres ≥1/16 in some epidemic foci such Angola (37) or Southern Sudan (38), seropositive individuals are currently not treated by control programmes in most endemic areas where the prevalence of disease is lower (11). Nevertheless, several studies have shown that parasite DNA could be detected in these subjects (39–42) and that they were able to maintain their serological status sometimes over very long periods (32). However, the infection status of these subjects has been questioned because (i) the CATT is known to lack specificity (11) and (ii) molecular characterization of these subjects required highly sensitive PCR, targeting repeated sequences that are not specific to *T. b. gambiense* but of *Trypanozoon* (43). Thus, it is difficult to rule out the fact that the observed results could be attributable to cross-reactions with other diseases or repeated exposure to tsetse flies infected by animal trypanosomes such as *Trypanosoma brucei brucei* (44). In a recent study, however, the highly specific immune trypanolysis test (TL) (45), which detects Litat 1.3 and Litat 1.5 variable surface antigens specific for *T. b. gambiense*, was applied to CATT seropositive subjects identified during medical surveys in both HAT active and historical HAT foci (46). Results showed that a fraction of the CATT seropositive subjects were TL positive and that the percentage of TL seropositive subjects was correlated with disease incidence reaching almost 80% in the most active focus of Guinea (44). However, there were no TL-positive cases where HAT transmission was not observed such as in the historical foci of Burkina Faso (despite the fact that 1·2% of the population was positive for CATT and tsetse flies and animal trypanosomiasis were still present). Finally, a follow-up study of patients with HAT before and after treatment and of seropositive subjects over time (Figure 1) indicated a rapid decrease in the CATT response in seropositive subjects negative to TL and a progressive decrease in the CATT response in patients after treatment associated with the disappearance of blood parasite DNA as measured by the TBR1/TBR2 diagnostic PCR. In contrast, all CATT seropositive subjects that were TL positive maintained a strong antibody response over time with parasite DNA being detectable at least once during the survey in half of them, suggesting that the presence of parasites in these subjects was responsible for the maintenance of the serological response directed against *T. b. gambiense* antigens (Ilboudo H, *et al,* in review). Thus, it appears that the seropositive status is a heterogeneous group owing to the following facts: (i) the CATT reaction may not be specific; therefore, this group may include false positives; (ii) these individuals may be in the very early phase of infection, such as those observed in the epidemic foci of Central Africa where approximately half of those individuals displaying CATT end titres of ≥1 : 16 went onto develop HAT in the following year (37,38); (iii) they may be asymptomatic carriers of parasites able to control blood parasitaemia to very low level for long periods of time, such as it is observed in West Africa in areas of lower endemicity (28). Taken together, the data presented above indicate that a wide range of outcomes (summarized in Figure 2) may occur as a result of infection by *T. b. gambiense* with both variation in the progression of first-stage to second-stage disease and the existence of serologically positive but apparently aparasitemic subjects that seem to be able to control blood parasitaemia to very low levels undetectable by the classical parasitological methods over long periods of time.

### Trypanotolerance in human: a controversial issue

Our point of view is that human trypanotolerance is a central question that will need to be investigated further in the coming years to provide significant advances in the knowledge of the complex interaction that occur between *T. b. gambiense* and its human host. Whereas trypanotolerance was clearly shown to exist in some West African Taurine cattle (47) and different inbred mice displaying contrasting susceptibility to trypanosomes infection (48), infection of humans by *T. b. gambiense* is still widely considered as invariably fatal. Both old observations reviewed by Checchi *et al.* and Sternberg *et al.* (27,49) and recent data now provide a bulk of evidence arguing this is not the case. On the contrary, it appears that some individuals are able somehow to control *T. b. gambiense* infections, suggesting that trypanotolerance also occurs in human populations. This observation raises two main questions.

First, how should these individuals be managed by NCPs? A better characterization of these subjects is of crucial importance because these subjects could be asymptomatic carriers of parasites, thus favouring maintenance or re-emergence of HAT. It is thus important to determine the role played by these individuals in parasite transmission. It is noteworthy that chronic bovine experimental *T. b. brucei* infections during which parasites are almost undetectable in blood are still infective for tsetse flies (50), suggesting that low parasitemic infections are still important in transmission. Similarly, we recently infected a pig with a *T. b. gambiense* field isolate that gave an infection profile with very low blood parasitaemia (in the range of 50–100 trypanosomes/mL) only occasionally detectable by microscopy after parasite concentration using the mini anion exchange centrifugation technique that is considered as the most sensitive parasitological test available to date (51,52). Interestingly, it was also possible to infect tsetse flies by feeding on this animal, and surprisingly, parasite concentration estimates in the gut of these flies dissected 24 h after the infective blood meal were almost 10 times higher as those in the blood of the pig (Woumbou C., unpublished data). A first hypothesis to explain how tsetse flies become infected by feeding on hosts with such low parasitaemia would be that *Glossina* saliva components may act as attractants concentrating trypanosomes to the bite site, thus increasing transmission probabilities. Another hypothesis is that trypanosomes could be located in other tissues at cutaneous sites rather than in venous blood.

A second important question is related to the nature of the host response in these seropositive subjects that is apparently able to control infection. Indeed, to date, all studies have focussed on the host response observed in patients in the different stages of the disease or in comparison with healthy endemic controls, but nothing is known on the response developed by seropositive individuals. Whether trypanotolerance in human is related to intrinsic factors from the host or parasite virulence factors or a combination of both remains to be determined. However, understanding how an individual is able to naturally control infection by *T. b. gambiense* is a highly desirable goal because it could lead to the identification of new targets from the host or the parasite for therapeutic or prophylactic interventions.

## The Host Immune Response to Infection

### Lessons from mice

As extracellular parasites that are continually exposed to the host’s immune system, African trypanosomes have evolved sophisticated evasion mechanisms to survive in the chronically infected host. Well-documented mechanisms include antigenic variation of the variant-specific surface glycoprotein (VSG) and the induction of alteration in the host’s defence system. The host’s ability to control parasitaemia in African trypanosomiasis involves at least four known mechanisms: (i) antibody/complement lysis, (ii) antibody-mediated phagocytosis, (iii) innate immunity in terms of primate-specific trypanolytic complexes in human serum (discussed in subsequent section) and (iv) release of trypanotoxic molecules such as reactive nitrogen intermediate or reactive oxygen intermediates by macrophages (53). Most of what is known to date on the host immune response directed against African trypanosomes is derived from experimental studies carried out in mice. The first reason is that in such experimental models, it is possible to control for environmental, host or parasite heterogeneities and that it is possible to follow the evolution of the immune response along with the course of infection, which is obviously impossible in humans. The second reason is that laboratory rodents on different genetic backgrounds display various degrees of susceptibility to the disease, thus providing important models to analyse the host mechanisms involved in the control of the infectious process (48). The various animal model systems used have provided conflicting evidence regarding the immunological factors that influence the magnitude of resistance to African trypanosomes. However, the overall picture is that the host response requires the contribution of both VSG-specific B- and T-cell responses and a proper activation of the macrophage/monocyte phagocyte system to control infection. Type 1 cytokine responses (INF-γ, TNF-α), leading to macrophage activation to produce trypanotoxic NO (54), are observed during the early stage of infection in both susceptible and resistant mice. In resistant mice, however, the cytokine profile switches to a type 2 response (IL-4, IL-10) during the late/chronic stages of infection presumably restricting prolonged and exaggerated inflammatory responses that may lead to tissue damage and early mortality (55). On the contrary, early mortality in highly susceptible mice is caused by an excessive activation of the macrophage system, associated with an excessive production of INF-γ and a systemic inflammatory syndrome (56,57). Whereas the mechanism regulating the host immune response is not yet fully understood, recent studies indicate an important role of cross-talk between CD8^+^ NKT cells that have the potential to control the parasite via macrophage-dependent production of NO and regulatory T cells (Tregs) that downregulate NKT cells during progressive infections (58,59).

Cells of the macrophage lineage provide the first line of defence against infectious diseases and modulate downstream events that impact on the development of acquired immunity. As stated earlier, macrophage activation is one of the hallmarks of infection with the African trypanosomes. Interestingly, increasing evidence shows that macrophages are also the cells that are targeted by trypanosomes to interfere with the host response to establish infection, by several different mechanisms. Parasite-derived molecules such as soluble VSG or DNA are thought to have deactivating properties on macrophages (53), presumably via the Toll-like receptor signalling pathways. Another mechanism of macrophage subversion is the IgM anti-VSG-mediated phagocytosis of trypanosomes via CR3, which enhances the synthesis of disease-promoting TNF-α (60) and inhibits synthesis of parasite-controlling NO (61).

### How relevant are mice models for *Trypanosoma brucei gambiense* infection in human?

In contrast to the large amount of data from mice models, relatively few studies have been carried out in man. Whereas mice provide good disease models, one has to keep in mind that there are differences in the physiology of mice and humans, for example, the lack of trypanolytic complexes. Thus, observations made in mice may not be directly extrapolated to the infectious process occurring during the human disease. This is particularly true for *T. b. gambiense* HAT. Whereas mice are highly susceptible to *Trypanosoma congolense*, *T. b. brucei* and *T. b. rhodesiense*, infections with *T. b. gambiense* field isolates require the use of immuno-deficient mice or BALB/c mice previously immunocompromised by the injection of cyclophosphamide (62).

To date, and for ethical and logistic reasons, most studies in humans have focused on the comparison of blood and CSF cytokine levels in patients with HAT at different stages of the disease. In sera, cytokine/chemokine levels are poorly correlated with disease stage. Yet, slightly higher concentration of IL-8 (63,64) and to a lesser extent IL-6 (64) were found in early-stage patients compared with intermediate- or late-stage patients. In contrast, IL-10 levels were found to be highest in late-stage patients (although the difference was not significant) (64). In CSF, however, more important differences are observed, and CSF collected from late-stage patients are characterized by elevated levels of IL-6, IL-8, IL-10, IL-1b, MCP-1 and MIP1-a (63,64). This cytokine storm includes both inflammatory cytokines that result from the generalized meningoencephalitis occurring in late-stage patients along with anti-inflammatory cytokines such as IL-10 produced presumably in an attempt to control excessive inflammation. The observations that these cytokine/chemokines returned to normal levels after treatment make them interesting markers for CNS invasion or for the follow-up of patients after treatment. In contrast to *T. b. rhodesiense* HAT in which elevated plasma levels of IFN-γ are observed during early stages and then decline in late stages (65), no variation of IFN-γ levels were reported in *T. b. gambiense*. As INF-γ is known to be transiently expressed early during infection both in mice and in the closer model of infection of *T. b. rhodesiense* in the Vervet monkey (66), it is possible that the apparent observed absence of IFN-γ in *T. b. gambiense* HAT is related to the chronic nature of early-stage disease.

A major impairment in the study of cytokine responses in HAT resides in the fact that all studies were focused on patients with disease, i.e. to the response of susceptible individuals. It is thus impossible to infer what type of responses could be protective. As stated earlier, increasing data indicate that trypanotolerant individuals exist also in humans. Furthermore, the tools enabling their characterization, such as long-term follow-up, positivity to TL and molecular diagnostic methods, are becoming available. We thus believe that studies aimed at characterizing the immune response in such subjects will constitute the next significant step towards a better understanding of *T. b. gambiense* immune control mechanisms in its human host. In this regard, to our knowledge, only one study has included seropositive individuals (*n* = 9). Although the effects were too small to draw conclusive results, this study has highlighted the fact that the cytokine response in these individuals was different to both HAT patients and endemic controls (67).

## Human Genetic Variation and Susceptibility to Infection

### Genetic basis for resistance in mice and cattle

Extensive work with experimental rodent models and crosses between trypanotolerant and susceptible cattle have demonstrated that the host genotype has a major impact on the progression of trypanosome infections (68). Three quantitative trait loci (QTL) controlling resistance to *T. congolense* infections in mice have been identified on chromosomes 17, 5 and 1 and designated *Tir1*, *Tir2* and *Tir3*, respectively (69). Fine mapping (70) has further improved the resolution of these QTLs and enabled the separation of Tir3 into three different loci (*Tir3a*, *Tir3b* and *Tir3c*). Whereas the relevant genes in these loci are presently unknown, there are several strong candidates: TNF-α gene (*Tir1*); IL-10 gene and Cypr2 (*Tir3b*); and possibly Cypr3 (*Tir2*). It is noteworthy that both Cypr2 and Cypr3 are IL-10 regulatory genes. Similarly, work on the bovine disease (71) has also identified a number of QTLs according to the phenotype analysed (parasitaemia, anaemia, body weight). Although loci identified in cattle are much larger and do not fully overlap with mice QTLs, it is interesting to note that the IL-10 and TNF-α genes are also encompassed within susceptibility loci in cattle (72).

### Cytokine polymorphisms

To our knowledge, only two studies aimed at studying human genetic factors influencing infection by *T. b. gambiense* have been carried out in Côte d’Ivoire and Democratic Republic of Congo. Both focused on the analysis of polymorphisms in cytokine genes with the development of HAT. These studies showed, on the one hand, a significant association with polymorphisms located in the IL-6 and IL-10 genes and a decreased risk of developing HAT and, on the other hand, a significant association between polymorphisms located in the IL-1*a* and TNF-*a* genes and an increased risk of developing the disease (73,74). However, significant association results were quite weak, indicating either that genetic variation at these loci does not play a major role in controlling the infection process or the existence of heterogeneity in the phenotype used for the analysis. However, the authors looked at the infected vs. uninfected status and did not differentiate between the different disease stages. As mentioned in the first part of the review, first-stage disease is likely to be very heterogeneous, comprising individuals that will rapidly evolve to the second stage, individuals that may remain in first stage for years or even individuals that will eventually self-cure (Figure 2). This may thus have impaired the statistical power to detect genetic association.

### Innate response candidate genes

In humans, the first lines of defence against trypanosomes are linked with components of the trypanolytic factor (TLF-1) that is lytic to almost all African trypanosomes except *T. b. rhodesiense* and *T. b. gambiense* (75). Human TLF-1 contains two primate-specific proteins, apolipoprotein L-1 (APOL1) and haptoglobin-related protein (HPR), as well as apolipoprotein A-1 (APOA1) that thus constitute important host components interacting with trypanosomes. APOL1 is the lytic protein forming anionic pores in the lysosomal membrane of the parasite (76,77). Resistance of *T. b. rhodesiense* to TLF-1 is attributable to the SRA protein, which has a high affinity to the SRA-interacting domain (SID) of the APOL1 protein and prevents trypanosome lysis (76,77). *SRA* is absent in *T. b. gambiense*, and the mechanism of resistance in this species seems to be linked to a low expression of the parasite Hb/Hp receptor that reduces uptake of APOL1 by *T. b. gambiense* compared with *T. b. brucei* and *T. b. rhodesiense* (78). Few studies on HAT have yet looked into the genetic variation of TLF-1 genes, whereas obviously variation in these key components of the innate immunity against trypanosomes may render the host more or less susceptible to infection. This is illustrated by the finding of a man infected by *Trypanosoma evansi* in India and who was subsequently found to be deficient in APOL1 (79). Furthermore, it was recently shown that two sequence variants located in the APOL1-SID domain were associated with the development of kidney disease in African Americans, suggesting that these polymorphisms were selected in Africa by trypanosomes. Consistent with this view, plasma from these APOL1-variants was able to lyse *T. b. rhodesiense* but not *T. b. gambiense* parasites *in vitro* (80). This suggests that these APOL1 polymorphisms may be major factors involved in determining human susceptibility to *T. b. rhodesiense* infection but could be less important for *T. b. gambiense* owing to the absence of the SRA gene. However, in the case of *T. b. gambiense*, a hypothesis could be that the interaction between trypanosomes and APOL1 occurs in a different manner, possibly for example through the modulation of APOL1 or APOL1-associated protein expression levels. In this regard, it is worth noting that APOL1 gene expression was shown to be upregulated by multiple pro-inflammatory signalling molecules including IFN-γ (81) and TNF-α (82). Alternatively, it could be that *T. b. gambiense* interacts with APOL1 at different sites of the protein. In this regard, it was found that the APOL gene family has rapidly evolved in simian primates, and signatures of positive selection in APOL1 have been found both inside and outside of the SRA-interacting domain (83). Further sequencing is now required to identify APOL1 genetic variants in the African populations living in *T. b. gambiense* endemic areas in order to test them for association with susceptibility to infection.

### The need for non-hypothesis-driven approaches

A limitation restricting the study of host genetics in HAT is that there has been a reliance upon hypothesis-driven approaches owing to the limited sample sizes available. Indeed, genome-wide association studies require the inclusion of thousands of subjects, and linkage analysis requires a large set of multi-case families. Because HAT prevalence is usually low and affected populations are usually in remote areas, it is difficult to build large enough cohorts to carry out such genome-wide genetic analysis. One strategy to overcome the limitation is to use genome-wide gene expression analysis to identify new candidate genes for which genetic association studies can be undertaken. Setting up such approaches in the future will be important as it could lead to the identification of human genes that control trypanosome infection. Furthermore, transcriptomic approaches have the potential to identify novel biomarkers of disease progression (first stage/second stage) or of trypanotolerance that could be useful in improving HAT diagnosis.

## Perspective for Future Work and Expected Outcomes

Whereas it is becoming increasingly clear that human infections by *T. b. gambiense* are not invariably fatal but in contrast result in a variety of infection outcomes, little attention has been paid to individuals that appear to be able to control infection. The nature of the immune response directed against trypanosomes in these subjects and the role they play in transmission are currently a black box that impairs our comprehension of both host–parasite interactions and disease epidemiology. Tools such as the trypanolysis test are now becoming available to differentiate between false and true positives of the CATT (46). Combined with the new molecular diagnostics methods (43) and follow-up of seropositive subjects, these tools will contribute to a better characterization/evaluation of trypanotolerance in human populations and will motivate future research on this important topic. Results of such research have important applications. First, in terms of disease control strategies: how should seropositive subjects be managed by NCPs? Should they be treated? These are questions that will need to be addressed if we are to achieve sustainable control of HAT. Furthermore, the existence of asymptomatic carriers of the parasite that are currently missed by medical surveys supports the idea that vector control campaigns aiming at lowering human–tsetse contacts in endemic areas may be required in addition to medical surveys to interrupt *T. b. gambiense* transmission. Second, understanding the biological mechanisms involved in human trypanotolerance should greatly help in the identification of new diagnostic, therapeutic or prophylactic targets. Whether susceptibility to *T. b. gambiense* infection is controlled by genetic factors from the host or by the parasite’s virulence will require further studies in which both parasite and host genetic diversity will have to be evaluated. The fact that it is now possible to genotype parasites by microsatellites directly from biological samples (84) should greatly help in identifying parasite genetic diversity in seropositive subjects. A limitation of immunological studies in man is that it is impossible to analyse the response along the course of infection and that events occurring early during the infection process are inaccessible. On the contrary, genetic factors are stable in time; thus, identification of genetic association within host genes may enable us to pinpoint specific molecules or pathways involved in the control of infection that could have been missed by immunological studies. Both immunological and genetic analyses of cohorts of well-characterized seropositive subjects and patients in first-stage or second-stage disease from Guinea and Côte d’Ivoire are currently underway and will provide, we hope, new insights into the mechanisms of trypanotolerance in humans in the near future. Such work should also be extended to other endemic areas such as Central Africa in order to provide a better view of trypanotolerance in HAT. Ideally, a survey of local wild fauna and domestic animals should also be carried out in these foci to evaluate their role in maintaining historical foci of disease.
